# A Clustering-Oriented Closeness Measure Based on Neighborhood Chain and Its Application in the Clustering Ensemble Framework Based on the Fusion of Different Closeness Measures

**DOI:** 10.3390/s17102226

**Published:** 2017-09-28

**Authors:** Shaoyi Liang, Deqiang Han

**Affiliations:** MOE KLINNS Lab, Institute of Integrated Automation, School of Electronic and Information Engineering, Xi’an Jiaotong University, Xi’an 710049, China; shaoyi.liang2@stu.xjtu.edu.cn

**Keywords:** clustering, clustering ensemble, closeness measure, geometric distance, neighborhood chain

## Abstract

Closeness measures are crucial to clustering methods. In most traditional clustering methods, the closeness between data points or clusters is measured by the geometric distance alone. These metrics quantify the closeness only based on the concerned data points’ positions in the feature space, and they might cause problems when dealing with clustering tasks having arbitrary clusters shapes and different clusters densities. In this paper, we first propose a novel Closeness Measure between data points based on the Neighborhood Chain (CMNC). Instead of using geometric distances alone, CMNC measures the closeness between data points by quantifying the difficulty for one data point to reach another through a chain of neighbors. Furthermore, based on CMNC, we also propose a clustering ensemble framework that combines CMNC and geometric-distance-based closeness measures together in order to utilize both of their advantages. In this framework, the “bad data points” that are hard to cluster correctly are identified; then different closeness measures are applied to different types of data points to get the unified clustering results. With the fusion of different closeness measures, the framework can get not only better clustering results in complicated clustering tasks, but also higher efficiency.

## 1. Introduction

Clustering is an important topic in machine learning, which aims to discover similar data and group them into clusters. Various clustering algorithms have been proposed and widely used in different areas such as sensor networks [1,2,3,4], image processing [5,6,7], data mining [8,9,10], text information processing [11,12], etc.

In classical clustering algorithms, the centroid-based methods, density-based methods and connectivity-based methods are the most commonly used in practice (such a categorization is according to the different cluster models employed). The most well-known clustering methods include the *k*-means [13], DBSCAN [14], CURE (Clustering Using REpresentatives) [15], etc. They respectively belong to the three aforementioned categories. There are also many recent works focused on improving the performance of the classic clustering schemes [16,17,18,19], or exploiting novel clustering methods using different closeness measures [20,21,22,23].

The effectiveness of clustering methods, to a great extent, is determined by the closeness measure between data points or clusters. In most classical methods, only the geometric distance is used to measure the closeness between data points, and the closeness between clusters is based on the closeness between their representative data points.

However, the distance-based metrics only focus on the geometric closeness. Although these metrics are appropriate for clustering tasks where the data points’ distribution conforms to some strong assumptions, e.g., regular shapes and uniform density, they perform not so well in complicated situations. For example, in *k*-means and its related methods, partitions are formed based on the distance between each data point and each centroid. Such partitioning rule will bring incorrect results when data points belonging to a given cluster are closer to the centroids of other clusters than to the centroid of the given correct cluster [24]. In DBSCAN, the clustering performance depends on the two parameters defining the neighborhood size and density threshold, which are based on some geometric distances [25]. Since the threshold is predefined and fixed, DBSCAN will generate incorrect results, if the densities of the data points in different clusters are varying. In existing agglomerative hierarchical clustering methods [26], sub-clusters are merged according to the closeness measures such as the single linkage and the complete linkage [27], where the closeness is determined by the pairwise geometric distances between inter-cluster representative data points. Due to the similar reasons that cause problems in the aforementioned methods, these agglomerative hierarchical algorithms usually work well only for the spherical-shaped or uniformly-distributed clusters [24].

The limitations of the traditional closeness measures in clustering have attracted much attention, and thus, many approaches using different closeness definitions have been proposed to address the aforementioned problems. One branch of methods uses the clusters’ probability distribution information and its closeness definition. For example, Lin and Chen [28] proposed the cohesion-based self-merging algorithm, which measures the closeness between two sub-clusters by computing and accumulating the “joinability” value of each data point in the two clusters. Dhillon et al. [29] used a KL divergence-based [30] clustering scheme to cluster words in the document categorization. KL-divergence is a measure of “distance” between two probability distributions (it is not a true distance metric because it is not symmetric, and it violates the triangle inequality). In the scheme, a word is assigned to a cluster if the distribution of this word has the smallest “distance” (measured by KL divergence) to the weighted sum of the distributions of the words in that cluster. Similar ideas can be seen in [31,32]. Heller et al. [33] and Teh et al. [34] used Bayes rule in a hierarchical clustering scheme to guide the merging process, where each pair of the clusters is assigned a posterior probability based on the Bayesian hypothesis test and the two clusters with the highest posterior probability are merged. By incorporating the distribution information, these methods are more robust to outliers and can deal with data with arbitrary shapes. However, users must know the clusters’ probability density functions (pdf) before running the algorithms. Another branch of refined clustering methods are based on the graph theory. Karypis et al. [24] proposed the algorithm of chameleon, where a graph based on *k*-nearest neighbors is constructed and then cut into sub-clusters. The relative inter-connectivity and the relative closeness are used to determine the closeness between sub-clusters, and the algorithm achieves good results in finding clusters with arbitrary shapes. Similarly, we can see in [35,36], and more recently in [37], that Zhang et al. proposed an agglomerative clustering method where a structural descriptor of clusters on the graph is defined and used as clusters’ closeness measure. The properties of graph theory make it very appropriate to describe clustering problems, and the methods based on graph theory perform well in dealing with clusters with arbitrary shapes. However, we find that these methods often fail to adapt to clustering tasks with very different clusters’ densities, although they use new closeness measures. In the two branches of refined clustering methods above, different kinds of closeness measures have been proposed to address the problems caused by the geometric distance-based closeness measures. These new measures do not focus on the geometric closeness alone, and they achieve success in many clustering tasks. However, they still have their own limitations. In particular, as mentioned above, these closeness measures either ignore the density information or need strong a priori information.

Therefore, in this paper, we first focus on designing a more comprehensive closeness measure between data points to substitute the traditional geometric distance-based closeness measures in clustering algorithms. The new measure is called the Closeness Measure based on the Neighborhood Chain (CMNC), where the neighborhood chain is a relationship established between two data points through a chain of neighbors. By substituting their original closeness measures with CMNC, many simple clustering methods can deal with the complicated clustering tasks with arbitrary clusters shapes and different clusters densities.

Prior to ours, there were some recent works in the literature that also utilized the *k*-nearest neighbors relationship in clustering problems. For example, Liu et al. [38] proposed a clustering algorithm named ADPC-KNN (Adaptive Density Peak Clustering *k*NN), where they modified the density peaks’ clustering [39] by using the distribution information of *k*-nearest neighbors of a data point to calculate its local density. Sur et al. [40] proposed a clustering method that forms a cluster by iteratively adding the cluster’s nearest neighbor into that cluster (a threshold is defined determining whether this nearest neighbor can be added into the cluster). In a series of work proposed in [41,42,43,44], Qiu et al. used an algorithm called nearest neighbor descent and its several modifications to organize the data points into a fully-connected graph “in-tree”, and the clustering results can be obtained after removing a small number of redundant edges in the graph. In the nearest neighbor descent algorithm, each data point “descends” to (links to) its nearest neighbor in the descending direction of density. Other similar works utilizing the *k*-nearest neighbors in clustering can be seen in [45,46].

In the above cited works, the neighborhood relationship is used in many ways and resolves different problems in clustering. However, our work presented in this paper is different from the existing methods. In our work, the neighborhood relationship is used to construct a pair-wise closeness measure between two data points, which incorporates not only the connectivity, but also the density information of data points.

The work in this paper is an extension of our previous preliminary work in [47], where the basic concepts of CMNC were preliminarily proposed. In this paper, we provide more detailed definition and analysis about CMNC. Furthermore, based on CMNC, we also propose a clustering ensemble framework that combines different closeness measures. Due to the involvement of neighborhood relationships, the computational cost of CMNC is relatively high. In the proposed framework, we use different closeness measures (CMNC and Euclidean distance) for different data points and get the unified clustering results. In this way, we are able to limit the use of CMNC to the “least required” number of data points to get the correct clustering results. Therefore, based on the proposed framework, we can get better clustering results and, at the same time, higher efficiency.

The rest of the paper is organized as follows. Section 2 introduces the basics of the traditional clustering methods and their limitations. In Section 3, the neighborhood chain is introduced, and CMNC is proposed. The performance of several clustering methods whose closeness measures are substituted with CMNC is provided. The clustering ensemble framework based on different closeness measures is proposed and tested in Section 4. Section 5 concludes this paper.

## 2. Traditional Clustering Methods and Their Limitations

In this section, we briefly recall some representative clustering methods. We focus on their closeness measures and discuss why these measures might cause problems.

### 2.1. Centroid-Based Clustering Methods

In the centroid-based clustering methods, e.g., the *k*-means [13] and the *k*-medoids [48], the closeness between a data point and the existing clusters’ centroid (or medoid) determines to which cluster the data point will be assigned. The clusters’ centroids are iteratively updated by minimizing the mean square distance of the data points to their assigned cluster’s centroids. In such a procedure, the closeness between a data point and a cluster’s centroid is measured by the geometric distance alone, which might cause problems.

**Figure 1:**
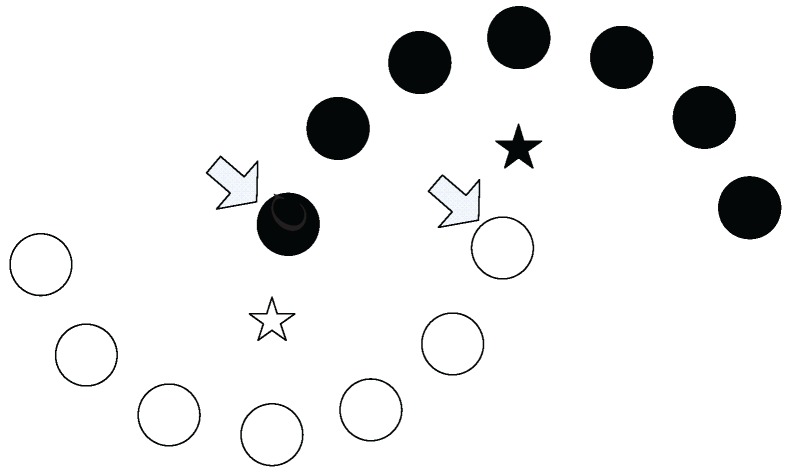
Illustration of problem in the centroid-based clustering method.

For example, in Figure 1, two clusters are represented by the hollow and the solid dots. The hollow star and the solid star are the centroids of the two clusters, respectively. In this case, the data points marked with arrows are geometrically closer to the centroid of the other cluster than to the centroid of their own cluster. Therefore, using such closeness measure, the clustering algorithm will bring incorrect clustering.

### 2.2. Density-Based Clustering Methods

DBSCAN is a representative density-based clustering method. It needs two predefined parameters eps and minpts, which respectively determine the closeness threshold and the minimum number of the data points to form a cluster. In DBSCAN, clusters are formed by the data points in the geometrically-dense regions [25]. The data points that are density-connected or density-reachable to each other will be assigned to the same cluster [14]. The parameters eps and minpts are used to determine whether a group of data is “dense” enough, or can be density-connected to each other. DBSCAN can achieve good performance when dealing with clusters with arbitrary shapes. However, the performance of DBSCAN is very sensitive to the parameters’ selection. Moreover, it may fail when the densities of the clusters are not concordant, even if the parameters are finely selected.

**Figure 2:**
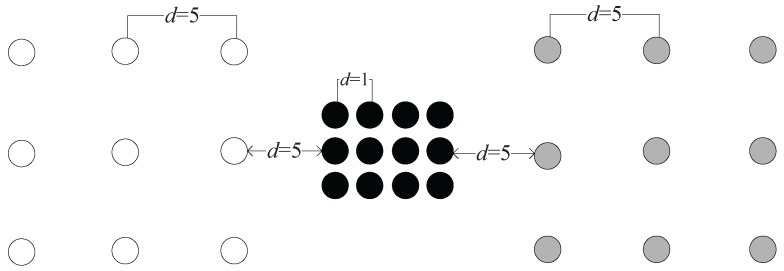
Illustration of the problem in DBSCAN method.

For example, in Figure 2, the hollow, the black solid and the gray solid dots are three different clusters. The densities of the clusters are different: data points in the black cluster are densely distributed, while those in the other two clusters are more sparsely distributed. In such a case, DBSCAN will fail to find all three clusters no matter how the closeness threshold is set. The reason for this problem lies in that the closeness threshold based on the geometric distance is predefined and is not adaptable to the change of clusters’ densities.

### 2.3. Connectivity-Based Clustering Methods

**Figure 3:**
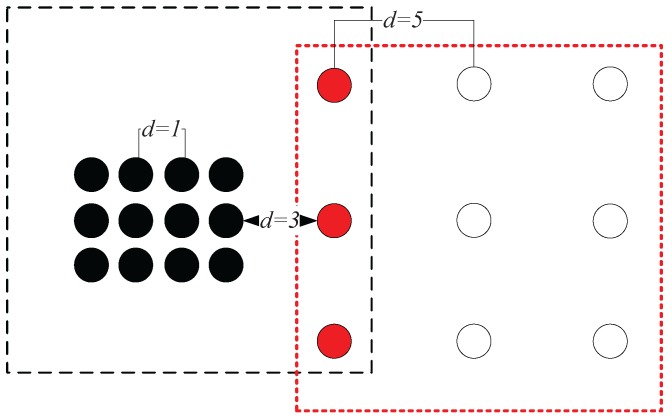
Illustration of the problem in the traditional agglomerative hierarchical methods.

Traditional agglomerative hierarchical clustering methods build cluster models based on distance connectivity. In these methods, all the data points are regarded as different clusters in the initial step. They are then iteratively merged according to the closeness measures between clusters. Although there are various methods to quantify the closeness between two clusters [26], these quantifications are mainly based on the geometric distance between two representative data points in the two clusters, and these methods mainly differ in how to choose the representative data points. For example, in the single-link method [49], the closeness between two clusters is represented by the distance between the geometrically closest pair of data points that respectively belong to the two clusters. CURE [15] is another famous hierarchical algorithm, where the clusters are not represented by a single data point, but a group of well-scattered representative data points. However, its closeness between two clusters is still measured by the geometric distance between the two clusters’ representative data points sets, which is essentially the same as other hierarchical methods. Therefore, generally speaking, these agglomerative hierarchical methods all suffer from the problems caused by their geometric distance-based closeness measures. For example, in Figure 3, the true cluster number is two. Assume that in the last iteration of the merging procedure, there are in total three clusters left. Using the aforementioned closeness measures for clusters, the red solid cluster will be merged with the black solid cluster because these two clusters are the closest according to the geometric distance metrics. However, the red cluster is apparently more likely to belong to the same cluster with the hollow cluster from the intuitive point of view.

In summary, quantifying the closeness is the key step in clustering algorithms, which determines the assignment of data points and the merging of clusters. The limitations of different categories of clustering methods discussed above are mainly caused by their closeness measures that only emphasize the geometric distances. Therefore, in order to resolve such problems, we propose a more comprehensive measure to quantify the closeness between data points.

## 3. Measuring Closeness between Data Points Based on the Neighborhood Chain

As discussed in Section 2, using the geometric distance alone to measure the closeness might cause problems. The main reason lies in that under the geometric distance metrics, the closeness between data points is fully determined by the positions of the two points being measured in the feature space, and the influence of any other surrounding data points is ignored. However, in many cases, being geometrically close does not necessarily mean that two data points are more likely to belong to the same cluster.

In this section, we propose a Closeness Measure based on the Neighborhood Chain (CMNC) that quantifies the closeness between data points by measuring the difficulty for one data point attempting to “reach” another through a chain of neighbors. The difficulty is measured by two quantifications called the neighborhood reachability cost and the neighborhood reachability span. Under such a closeness measure, a data point can reach another data point at a low cost as long as they belong to the same cluster, while a data point costs much more to reach another if they belong to different clusters.

Note that the terms “reach” and “reachability” have also appeared in DBSCAN and OPTICS (Ordering Points To Identify the Clustering Structure) [50], describing whether two data points are density connected based on the geometric distance alone. However, in this paper, the “neighborhood reachability” is defined based on the neighborhood relationship between two data points.

### 3.1. Neighborhood Chain

The neighborhood chain contains a series of data points, including a start point and an end point. Each data point in the chain (except the start point) is one of the *k* nearest neighbors of its precedent data point. Before giving the formal definition of the neighborhood chain, we first use an example to intuitively illustrate how a chain is established.

**Figure 4:**
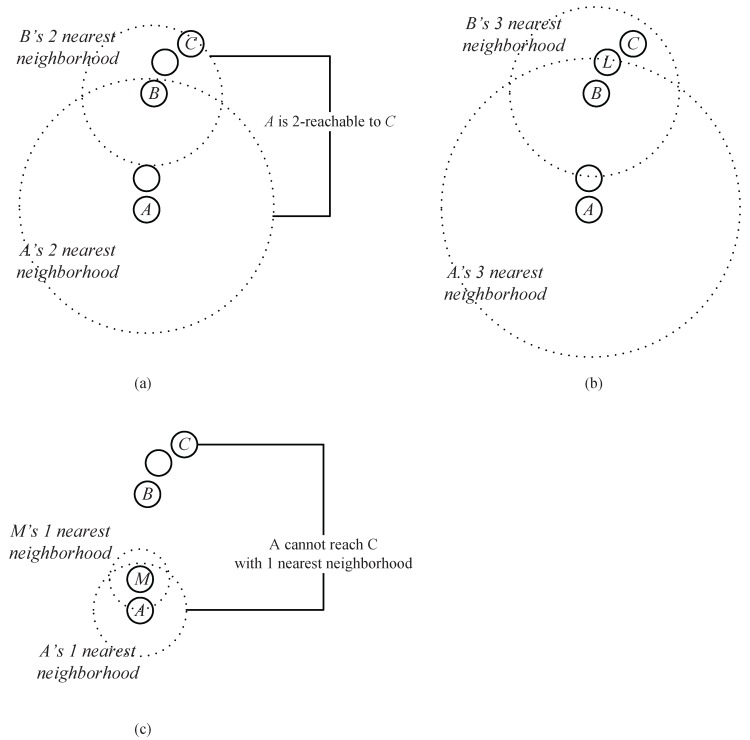
(**a**) *A* can reach *C* based on two nearest neighbors. (**b**) *A* can also reach *C* based on three nearest neighbors. (**c**) *A* cannot reach *C* based on one nearest neighbor.

**Example** **1.** As shown in Figure 4a, assume that A, B and C are the data points in a dataset. Obviously, B is in the two nearest neighbors of A, and C is in the two nearest neighbors of B. Thus, through an intermediate data point B, a chain from A to C based on the two nearest neighbors is established. We say that C can be reached by A via the chain of two nearest neighbors through B.

As shown in Figure 4b, *B*is also in the three (or more) nearest neighbors of *A*, and *C* at the same time is in the corresponding number of the nearest neighbors of *B*, which means that the chain from *A* to *C* based on three (or more) neighbors can also be established. However, the chain established based on the two neighbors takes a lower cost (which means that the required neighbor’s number is less).

Actually, in Figure 4, two nearest neighbors comprise the minimum requirement to establish a chain from *A* to *C* (Figure 4c shows that the chain from *A* to *C* cannot be established based on one nearest neighbor). Therefore, we say that *A* can reach *C* through a neighborhood chain with two nearest neighbors, or *C* is two-reachable from *A*.

The formal definition of the neighborhood chain is as follows. Assume that $\Omega \subseteq {ℜ}^{n}$ is a dataset and A,C∈Ω. Let f(A,C,Ω) be a positive integer that makes a set of data points $\left\{A,M_{1},M_{2},\ldots ,M_{q},C\right\}$ in Ω satisfy:

(1) $$\left\{\begin{array}{c} M_{1}\in Neighbors\left(A,f\left(A,C,\Omega \right)\right)\\ M_{i}\in Neighbors\left(M_{i-1},f\left(A,C,\Omega \right)\right),1<i\leq q\\ C\in Neighbors(M_{q},f\left(A,C,\Omega \right)) \end{array}\right.$$

where Neighbors(·,f(A,C,Ω)) represents the set containing a data point and its f(A,C,Ω) nearest neighbors. If such an integer f(A,C,Ω) exists, we say that the neighborhood chain from *A* to *C* can be established.

In the given dataset Ω, f(A,C,Ω) can take different values to establish different chains from data point *A* to *C*. For example, in Example 1 shown above, f(A,C,Ω) can be 2, 3 or 4 (or even more), which respectively can establish the chain *A*-*B*-*C*, *A*-*L*-*C*, or *A*-*C*. Therefore, we define:

(2) R(A,C)=min(f(A,C,Ω))

as the required neighbor’s number to establish the neighborhood chain from *A* to *C*. In the rest of this paper, the neighborhood chain refers to the chain established based on the required neighbor’s number.

When the required neighbor’s number is determined, the corresponding data points $\left\{M_{1},M_{2},\ldots ,M_{q}\right\}$ that satisfy Equation (1) are called the intermediate point in the chain from *A* to *C*. In Figure 4a, R(A,C)=2, and *B* is an intermediate point from the data point *A* to *C*.

**Figure 5:**
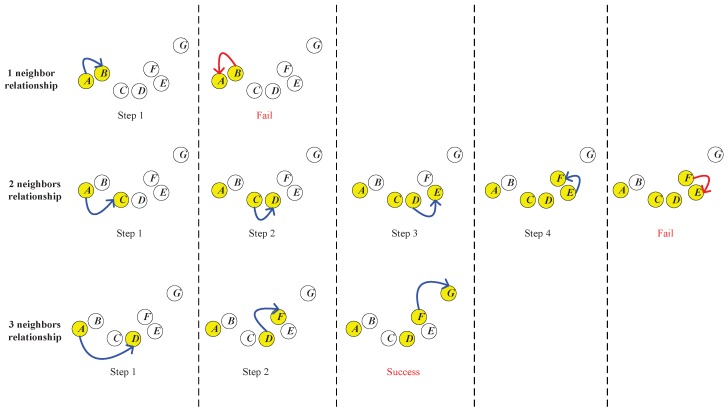
“Trial and error” process to determine the required neighbor’s number.

In practice, the required neighbor’s number and the corresponding intermediate points can be determined through a “trial and error” process. Such a process can be illustrated by Figure 5.

As shown in Figure 5, *A*, *B*,...,*G* are seven data points in a dataset. Now, we would like to establish a neighborhood chain from *A* to *G* and determine the required neighbor’s number R(A,G). First, we try the one nearest neighbor relationship. As shown in the first row of Figure 5, in Step 1, we search the nearest neighbor of the start point *A*, which is *B*, and add it into the chain. In Step 2, we continue to search the nearest neighbor of *B*. However, we find that *A* is *B*’s nearest neighbor, and *A* is already in the chain. Therefore, the searching process enters a loop, and the chain from *A* to *C* cannot be established.

Then, we start over to try the two nearest neighbors’ relationship. As shown in the second row of Figure 5, in Step 1, we find that *B* and *C* are both in the two nearest neighbors of the start point *A*, and we add *C* into the chain, because *C* is closer to the destination (the end point *G*). In Step 2, we continue to search the two nearest neighbors of *C*, which is newly added into the chain, and *B* and *D* are found. In this step, *D* is added into the chain because it is closer to *G*. In Steps 3 and 4, *E* and *F* are added into the chain sequentially. However, in Step 5, when we search the two nearest neighbors of the newly added *F*, we only find *E* and *D*, which are both in the chain already. Therefore, the searching process fails in this step, and the chain from *A* to *G* cannot be established.

As shown in the third row of Figure 5, we start over to try the three nearest neighbors’ relationship. In Step 1, we add *D* into the chain, because it is the closest to the destination (the end point *G*) in the three nearest neighbors of *A*. In Step 2, we find *C*, *E* and *F* in the three nearest neighbors of *D*, and we add *F* into the chain for the same reason. In Step 3, we find the end point *G* in the three nearest neighbors of *F*, which means that the chain from *A* to *G* is successfully established. Therefore, the three nearest neighbors comprise the minimum neighbor’s number required to establish the chain, which means that R(A,G)=3.

Along with the determination of R(A,G), the neighborhood chain from *A* to *G* is obtained, which is *A*-*D*-*F*-*G*. {D,F} is the set of intermediate points of the chain from *A* to *G*.

In practical applications, in the “trial and error” process to establish the neighborhood chain, we might encounter some situations where several data points in the neighborhood of a point (which is newly added into the chain) have the same distance to the end point we want to reach. In such situations, we just randomly choose one of these points to be added into the chain.

### 3.2. Quantifying the Difficulty to Establish a Neighborhood Chain

In this part, we define two quantifications of the difficulty in establishing a neighborhood chain, which are the neighborhood reachability cost and neighborhood reachability span.

**Figure 6:**
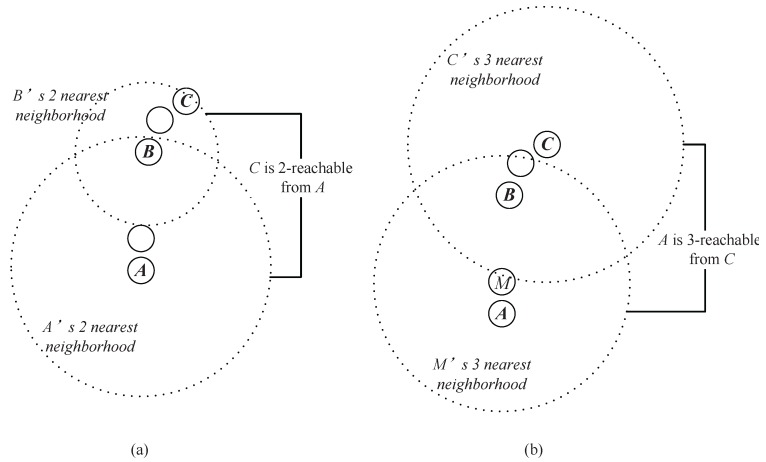
(**a**) The required neighbor’s number from *A* to *C* is 2. (**b**) The required neighbor’s number from *C* to *A* is 3.

Neighborhood reachability cost (NRC): The neighborhood reachability cost is designed based on the required neighbor’s number when establishing a chain from one data point to another. Note that the required neighbor’s number is usually not symmetric, i.e., the required neighbor’s number from a data point *A* to another data point *C* is usually different from that from *C* to *A* (as shown in Figure 6). We define a symmetric quantification:

(3) NRC(A,C)=f(max(R(A,C),R(C,A)))

as the neighborhood reachability cost when establishing a chain between *A* and *C*, where f(·) can be a function that is monotonically increasing on (0,∞). Obviously, the more neighborhood reachability cost needed, the more difficulty in establishing the chain.

In the rest of this paper, f(·) in Equation (3) is designated as the exponential function, because it can make the NRCvalue grow much faster than the *R* value grows, which will magnify the difference between the closeness value of two data points from the same cluster and that of two data points from different clusters.

In Equation (3), max{·,·} is used to select the bigger one out of R(A,C) and R(C,A) to make NRC(A,C) a symmetric quantification.

Neighborhood reachability span (NRS): Although using the geometric distance alone to measure the closeness between data points might cause problems as previously mentioned, it can still be used as a part of the closeness measure to depict in detail the difficulty in establishing a neighborhood chain. The neighborhood reachability span of a neighborhood chain quantifies the maximum span (distance) between the two adjacent intermediate points in a chain. ∀A,C∈Ω, if $\left\{M_{1},M_{2},\ldots ,M_{n}\right\}$ are the intermediate points in the chain from *A* to *C*, then there is:

(4) $$S\left(A,C\right)=\max_{}\left\{d\left(A,M_{1}\right),\ldots ,d\left(M_{n-1},M_{n}\right),d\left(M_{n},C\right)\right\}$$

where S(A,C) is the unidirectional span in the chain from *A* to *C*, and d(·,·) is the Euclidean distance between two intermediate points. The neighborhood reachability span of the chain between two data points *A* and *C* is defined as:

(5) NRS(A,C)=max{S(A,C),S(C,A)}

By selecting the bigger one out of the two unidirectional spans, the NRS is also a symmetric quantification.

### 3.3. Closeness Measure between Data Points Based on the Neighborhood Chain

The neighborhood reachability cost and the neighborhood reachability span are two parts that jointly quantify the difficulty to establish a neighborhood chain between two data points, and the difficulty in establishing the chain can be used to measure the data points’ closeness. The CMNC between any two data points *A* and *C* in a dataset is defined as:

(6) $$CMNC\left(A,C\right)=\frac{1}{NRC(A,C)\cdot NRS(A,C)}$$

A bigger CMNC value means that the chain between the two data points can be more easily established, which represents that the two points are closer, while a smaller CMNC represents the opposite. Strictly speaking, CMNC is not a distance metric since it violates the triangle inequality due to the use of the neighborhood relationship. However, using CMNC as a kind of closeness (similarity) measure, we can obtain more intuitive and rational closeness quantifications compared with using traditional closeness metrics based on the geometric distance alone in clustering tasks. The followings are two examples illustrating the computation of CMNC.

**Figure 7:**
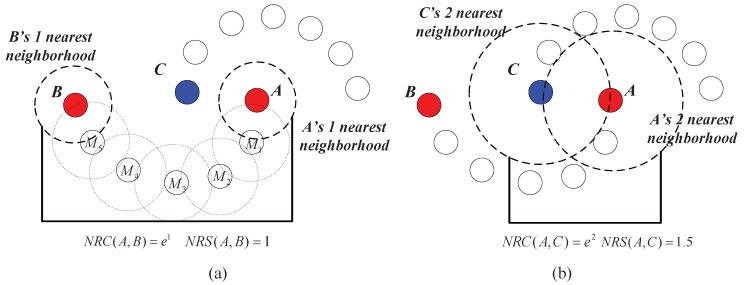
(**a**) Value of the closeness Measure based on the Neighborhood Chain (CMNC) between *A* and *B*. (**b**) Value of CMNC between *A* and *C*.

**Example** **2.** As shown in Figure 7a,b, assume that the distance between any two adjacent data points belonging to the same cluster is one (e.g., the distance between $M_{1}$ and $M_{2}$ is one) and the distance between the nearest pair of data points that belong to different clusters is 1.5 (i.e., the distance between A and C is 1.5).

In Figure 7a, we calculate the CMNC value between data points *A* and *B*. Note that $M_{1}$ is in the nearest neighborhood of *A*; $M_{2}$ is in the nearest neighborhood of $M_{1}$; and the relationship spread all the way to B. Therefore, R(A,B)=R(B,A)=1, and we have $NRC\left(A,B\right)=e^{\max\{RC(A,B),RC(B,A)\}}=e^{1}\;\approx \;2.72$. $M_{1}$ to $M_{5}$ are the intermediate points in the chain between *A* and *B*, and the distance between any two adjacent intermediate points is one. Therefore, we have NRS(A,B)=max{S(A,B),S(B,A)}=1. Then, CMNC(A,B)=1/[NRC(A,B)·NRS(A,B)]=0.37.

In Figure 7b, we calculate the CMNC value between data points *A* and *C*. Note that two nearest neighbors are needed to establish a chain between *A* and *C*, so we have $NRC\left(A,C\right)=e^{2}\approx 7.40$ and NRS(A,C)=1.5. Therefore, CMNC(A,C)=1/[NRC(A,C)·NRS(A,C)]=0.09.

In this example, we see that although the geometric distance between *A* and *B* is much longer than that between *A* and *C*, *A* and *B* is much “closer” than *A* and *C* using the CMNC measure.

**Figure 8:**
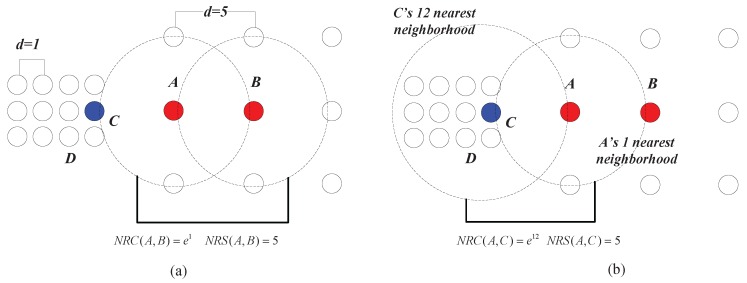
(**a**) CMNC value between *A* and *B* in clusters with different scales. (**b**) CMNC value between *A* and *C* in clusters with different scales.

**Example** **3.** As shown in Figure 8a, the chain between A and B can be established with one nearest neighbor, thus $NRC\left(A,B\right)=e^{1}$ and NRS(A,B)=5. In Figure 8b, C can be reached by A with one nearest neighbor, and thus, R(A,C)=1. However, the lowest cost that it takes for A to be reached by C is 12 nearest neighbors, which means that R(C,A)=12. Therefore, we have $NRC\left(A,C\right)=e^{12}$. This shows that although the geometric distance between A and C is equal to that between A and B (e.g., the Euclidean distances between A and C and that between A and B are both five in this case), the data points from two different clusters can be clearly separated using the CMNC measure ($CMNC\left(A,C\right)=1/\left(5e^{12}\right)\approx 1.2\times 10^{-6}$ is much smaller than $CMNC\left(A,B\right)=1/\left(5e^{1}\right)\approx 7.4\times 10^{-2}$).

In fact, in the case shown in Figure 8, the neighborhood reachability cost between any two data points that belong to the same cluster is always $e^{1}$ (e.g., $NRC\left(A,B\right)=NRC\left(C,D\right)=e^{1}$). This means that although the densities of the two clusters are different from the global point of view, the relationship between data points within each cluster, from the clusters’ local point of view, are very similar. In other words, this means that seeing from the individual cluster they belong to, the closeness between *A* and *B* is similar to that between *C* and *D*. In such a situation, the closeness of data points depicted by CMNC can adapt to different clusters’ “local density”, while traditional density-based methods like DBSCAN using geometric distance metrics alone can only deal with the clusters having the same density.

In the last part of this section, we give the algorithm to compute the CMNC value in pseudocode. The Algorithm 1 is as follows:
**Algorithm 1**Input: Start_Point=A, End_Point=C, k=1, Result_Set={A}, and the dataset Ω.Output: the value of CMNC(A,C).  S1: find Start_Point’s *k* nearest neighbors in Ω, and denote them as kNN_set.  S2: In kNN_set, find the point closest to End_Point, and denote it as temp_Point. If multiple points in kNN_set have the same distance to End_Point, choose one randomly, and denote it as temp_Point.  S3: If temp_Point==End_Point, GO TO S5. Otherwise, GO TO S4.  S4: If temp_Point is found in Result_Set, then set Result_Set = {A}, Start_Point=A, *k* = *k* + 1, and GO TO S1.   Otherwise, Result_Set=Result_Set∪temp_Point, Start_Point=temp_Point, and GO TO S1.  S5: If this is the first time entering S5, then R(A,C)=k, and S(A,C) equals the maximum distance between any two adjacent points in Result_Set. Set k=1, Result_Set={C}, Start_Point=C, End_Point=A, and GO TO S1.   Otherwise, R(C,A)=k, and S(C,A) equals the maximum distance between any two adjacent points in Result_Set. GO TO S6.  S6: Calculate $NRC\left(A,C\right)=e^{\max\{R(A,C),R(C,A)\}}$, NRS(A,C)=max{S(A,C),S(C,A)}, and CMNC(A,C)=11NRC(A,C)·NRS(A,C)NRC(A,C)·NRS(A,C).


### 3.4. Computational Complexity of CMNC

In the worst case, the computational cost to calculate CMNC(A,C) could be $O\left(n{\left(n-1\right)}^{2}\right)$. In the following, we will illustrate how the expression is obtained.

In order to quantify the computational cost when measuring the closeness between two data points (say *A* and *C*) with CMNC, we need to examine the computational cost of each component (NRC and NRS) of the CMNC measure.

Computational cost to calculate NRC(A,C): As shown in Equation (3), we need first to compute R(A,C) and R(C,A) before computing NRC(A,C). As illustrated in Section 3.1, we use a “trial and error” process to build the neighborhood chain from *A* to *C*. Assume that we need to try *t* times to successfully build the chain from data point *A* to *C* (which means that the chain is established on the *t*-th try, and R(A,C)=t), and in each try *i*, 0<i<t, we have added $m_{i}$ points into the (unaccomplished) chain before the establishing of the chain fails. In the *t*-th try, the chain is established; therefore, $m_{t}$ is the number of the intermediate points from *A* to *C*. Under such assumptions, we actually need to execute $\sum_{i=1}^{t}m_{i}$ times the nearest neighbor searching algorithm, where the distance between one data point (whose neighbors are to be found) and all other data points in the dataset will be computed. Therefore, the computational cost calculating R(A,C) can be expressed as $O(n\cdot \sum_{i=1}^{t}m_{i})$, where *n* is the number of data points. Similarly, we assume that $R\left(C,A\right)=t^{*}$ (which means that the chain from *C* to *A* is established on the $t^{*}$-th try), and in each try to establish the chain from *C* to *A*, $m_{j}^{*}$ ($0<j\leq t^{*}$) represents the number of data points added into the chain. The computational cost calculating R(C,A) can be expressed as $O(n\cdot \sum_{j=1}^{t^{*}}m_{j}^{*})$. Therefore, the computational cost calculating NRC(A,C) can be obtained by summing the cost of R(A,C) and R(C,A) and be expressed as $O\left(n\cdot \sum_{0<i\leq t,0<j\leq t^{*}}\left(m_{i}+m_{j}^{*}\right)\right)$.

Computational cost to calculate NRS(A,C): As shown in Equations (4) and (5), we need to compute the distance between each pair of the adjacent intermediate points in the chain from *A* to *C* and that from *C* to *A*. Therefore, under the assumptions in the previous paragraph, the computational cost calculating NRS(A,C) can be expressed as $O(m_{t}+m_{t^{*}}^{*})$.

The total computational cost to calculate CMNC(A,C) can be obtained by summing the cost of NRC(A,C) and NRS(A,C). In normal situations, $m_{t}$ and $m_{t^{*}}^{*}$ are much less than the data points number *n*; therefore, the cost of NRS(A,C) is negligible. The computational cost to calculate CMNC(A,C) can be expressed as $O\left(n\cdot \sum_{0<i\leq t,0<j\leq t^{*}}\left(m_{i}++m_{j}^{*}\right)\right)$.

The expression illustrates that the computational cost to calculate CMNC(A,C) is determined by the total execution times of the nearest neighbor searching algorithm in the establishing of the neighborhood chain from *A* to *C* and from *C* to *A*. Such an expression of the computational cost depends largely on the inner structures of the given dataset, and the selected start and end points; therefore, we can hardly use it to evaluate the average computational cost to calculate the CMNC value between any two data points in a dataset. However, we can still use it to estimate the highest possible cost to calculate CMNC(A,C) in the worst case. In the extreme situation, *t* and $t^{*}$ can both reach n−1, and $m_{i}$ and $m_{j^{*}}^{*}$ can also reach n−1. Therefore, in the worst case, the computational cost to calculate CMNC(A,C) could be $O\left(n{\left(n-1\right)}^{2}\right)$.

### 3.5. Substituting Closeness Measures in Traditional Clustering Methods with CMNC

In this part, we test the performance of the proposed closeness measure by substituting the geometric-distance-based closeness measures between data points in some clustering methods with CMNC. The methods for testing include the original versions of *k*-medoids, single-link, CURE and their CMNC-based versions (the closeness between the data points or between clusters in these methods is calculated with pairwise-distance-based measures; thus, it is easy to substitute these measures with CMNC). DBSCAN is also tested for comparison.

In the test of all the original version methods, Euclidean distance is used as the closeness measure. In the test of *k*-medoids and CMNC-based *k*-medoids, to exclude the impact of initial centers selection, we give them the same initial centers, where each center is randomly selected from one natural cluster. In CMNC-based *k*-medoids, the “distance” between data points and the centers is calculated with CMNC. In CMNC-based single-link method, the similarity of two clusters is represented by the CMNC value of their most similar (using CMNC measure) pair of data points. In CMNC-based CURE method, the clusters with the closest (using CMNC measure) pair of representative data points are merged in each iteration. The natural (true) clusters number is assigned to *k*-medoids, single-link, CURE and their CMNC-based versions as the input parameter.

**Figure 9:**
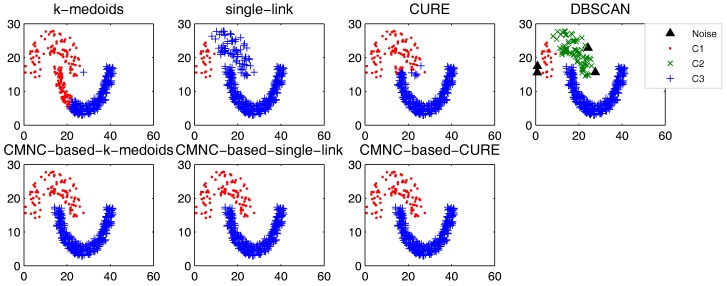
Comparison of clustering results on the “double-moon” dataset.

**Figure 10:**
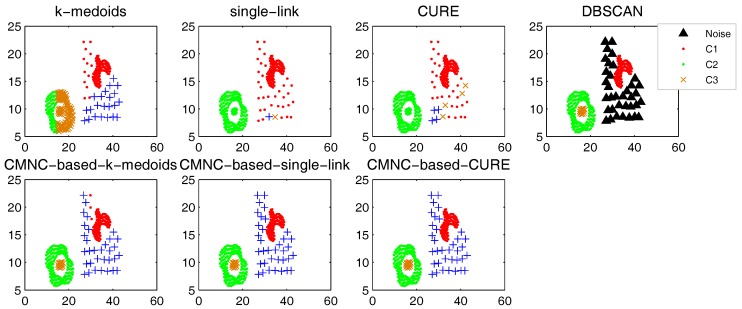
Comparison of clustering results on the “compound” dataset.

**Figure 11:**
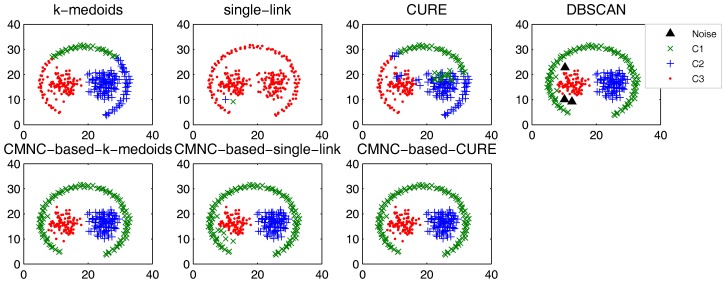
Comparison of clustering results on the “path-based” dataset.

The test results of the aforementioned methods on three datasets [51,52,53] are shown in Figure 9, Figure 10 and Figure 11. For DBSCAN, the shown results are the best results selected by traversing its parameters eps and minpts.

In Figure 9, there are two natural clusters, and the two clusters have different densities and twisted shapes. In Figure 10, four natural clusters can be found, in which two clusters have the shapes of concentric circles and the other two have very different densities. In Figure 11, there are three clusters in total, and two of them are spherically distributed. However, the third one that surrounds the two clusters makes the situation more complicated.

These datasets are difficult for the test using traditional clustering methods, and they fail to find all the clusters correctly as shown in Figure 9, Figure 10 and Figure 11. However, by substituting the closeness measures, the CMNC-based methods successfully find the correct clusters in all the tested datasets. In this test, DBSCAN can also handle the non-spherical clusters shapes. However, it cannot deal with the clusters having different densities. For example, in Figure 10, if a big eps is set, the two natural clusters in the upper-right corner will be considered as a whole cluster, while if a small eps is set, the data points in the sparsely-distributed cluster will all be considered as the noise.

We have also tested the clustering methods on some UCI datasets [54]. The Normalized Mutual Information (NMI) [55] and the Clustering Error (CE) [56] are used as the quantitative criterion for the performance evaluation of the tested methods.

NMI provides an indication of the shared information between a pair of clusters [55]. The bigger this NMI value, the better the clustering performance. For CE, obviously, a lower value is preferred. The test results are shown in Table 1 and Table 2. For *k*-medoids and CMNC-based *k*-medoids, the results shown are the average results of 20 runs. For CUREand CMNC-based CURE, the results shown are the best results found by traversing the needed parameters.

Note that the CE and NMI results of *k*-medoids and CMNC-based *k*-medoids methods are obtained by averaging the results of 20 runs, where their initial centers are chosen randomly, so we need further to implement a test of significance to validate that the results of CMNC-based *k*-medoids are significantly better than that of the original *k*-medoids method. The methodology we use in this paper is the *t*-test [57]. In the test, we assume that the CE and NMI results of each run of *k*-medoids and CMNC-based *k*-medoids come from two normal distributions that have the same variance. The null hypothesis ($H_{0}$) is that the mean of the CE (or NMI) results of CMNC-based *k*-medoids equals that of the original *k*-medoids method. On the contrary, $H_{1}$ represents that the mean values of the two groups of data are statistically different (under certain significance level). If $H_{0}$ holds, we have:

(7) $$\frac{\bar{X}-\bar{Y}}{S_{w}\sqrt{\frac{1}{n_{1}}+\frac{1}{n_{2}}}}∼t\left(n_{1}+n_{2}-2\right)$$

where $\bar{X}$ and $\bar{Y}$ respectively represent the mean of the CE (or NMI) results obtained by CMNC-based *k*-medoids and the original *k*-medoids methods, $n_{1}$ and $n_{2}$ respectively represent the number of instances in *X* and *Y* and:

(8) $$S_{w}=\sqrt{\frac{\left(n_{1}-1\right)S_{1n_{1}}^{2}+\left(n_{2}-1\right)S_{2n_{2}}^{2}}{n_{1}+n_{2}-2}}$$

where $S_{1n_{1}}^{2}$ and $S_{2n_{2}}^{2}$ respectively represent the variance (using Bessel’s correction) of the two sets of results.

If the observation of the *t*-statistic $t=\frac{\bar{X}-\bar{Y}}{S_{w}\sqrt{\frac{1}{n_{1}}+\frac{1}{n_{2}}}}$ falls into the rejection region, which means that $\left|t\right|\geq t_{\alpha /2}\left(n_{1}+n_{2}-2\right)$, then $H_{0}$ will be rejected, representing that the mean values of the CE and NMI results obtained by CMNC-based *k*-medoids are statistically different from those obtained by the original *k*-medoids method. The test results are shown in Table 3 and Table 4.

The results in Table 3 and Table 4 illustrate that we have sufficient reasons to reject the null hypothesis $H_{0}$ on all the tested datasets (under significance level α=0.1), which means that the results obtained by CMNC-based *k*-medoids are statistically better (lower under CE index and higher under NMI index) than those of the original *k*-medoids method.

Synthesizing the clustering results shown in Figure 9, Figure 10 and Figure 11 and the test results in Table 1, Table 2, Table 3 and Table 4, it can be concluded that, on the tested datasets, by substituting the closeness measures with CMNC, the CMNC-based methods can counter-act the drawbacks of the traditional methods and generate correct results in clustering tasks with arbitrary cluster shapes and different cluster scales. The results also show that the CMNC-based methods can work well for the tested real-world data and can achieve better performance (under CE and NMI indexes) than their original versions.

## 4. Multi-Layer Clustering Ensemble Framework Based on Different Closeness Measures

In previous sections, we proposed CMNC to deal with the problems brought by the closeness measures based on geometric distance and achieved good results in clustering tasks with arbitrary cluster shapes and different cluster densities. However, as shown in Section 3.4, the computational cost of CMNC is high due to the involvement of neighborhood relationships. Actually, in many simple clustering tasks, or for the “simple part” of some complicated tasks, the geometric distance-based closeness measures can also lead to satisfactory clustering results. They can handle these simple tasks with low computational cost, and they are also easy to implement. Therefore, in this section, we try to incorporate the advantages of CMNC and geometric distance-based closeness measures to deal with complicated clustering tasks with higher clustering accuracy and, at the same time, higher time efficiency.

In order to combine the two kinds of closeness measures, we propose a multi-layer clustering ensemble framework. In this framework, the data points that are hard to group into the correct cluster (we call them the “bad data points”, e.g., the data points in the overlapping regions of two non-spherical shaped clusters) can be identified. Thus (in prototype-based clustering schemes), we can apply CMNC only to these data points when calculating their closeness to the clusters’ centroids. In this way, the new framework can retain the low computational cost in simple clustering tasks, where only a few “bad data points” need to be dealt with; while in complicated cases, the new framework can achieve much better clustering accuracy than traditional ones and not much computational cost due to the selective application of CMNC.

**Figure 12:**
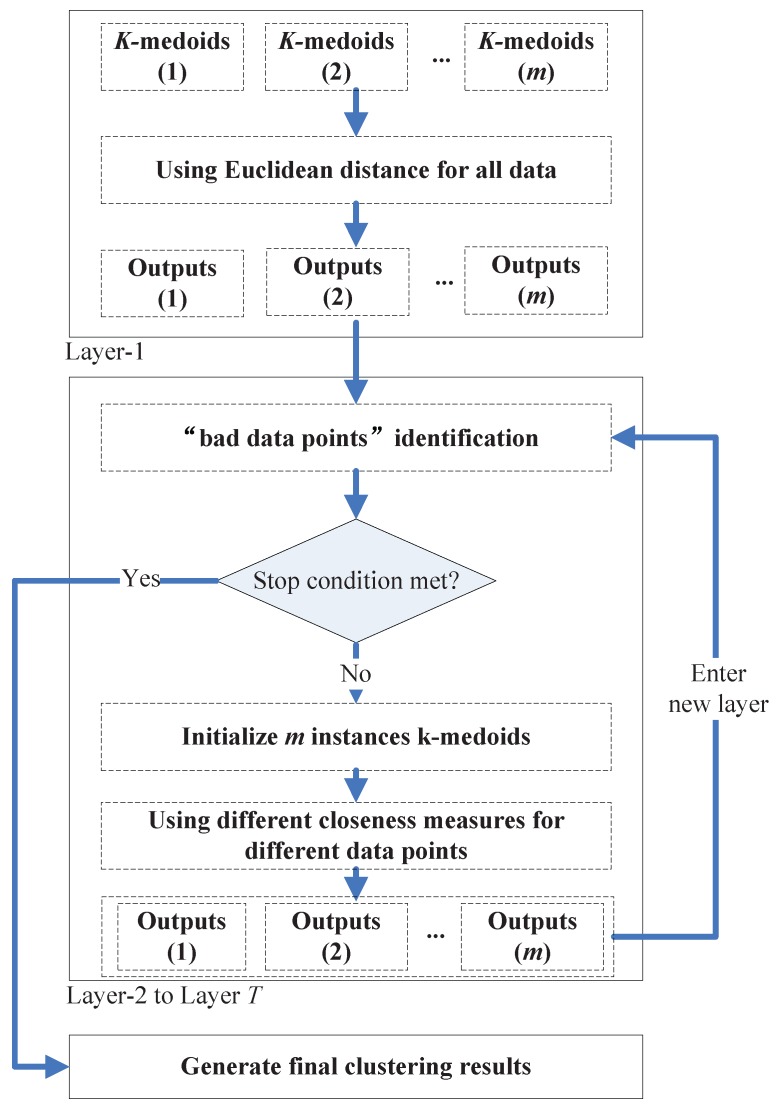
Illustration of the implementation of the proposed framework.

In the framework, a group of *k*-medoids algorithms with random *k* values ($2\leq k\leq k_{\max}$) and *k* initial centroids (in the rest of this paper, we will call them the “member clusterers”) run repeatedly from Layer-1 to Layer-*T*. In the first layer, Euclidean distance is used as the closeness measure of all pairs of data points. In the following layers, along with the execution of the “bad data points” identification algorithm on the previous layer’s output, some data points will be identified to be the “bad data points”, and CMNC will be used when calculating the “distance” between these “bad data points” and the clusters’ centroids in the member clusterers. The identification algorithm will be executed once in each new layer based on the outputs of the previous layer, and the iteration ends when no more “bad data points” (compared with the “bad data points” number found in the previous layer) are found in a certain layer, or when the user assigned maximum layer’s iteration number *T* is met. One additional *k*-medoids (or other clustering methods) will be executed based on the finally found “bad data points” and normal data points to generate the final clustering results. Figure 12 shows an illustration of the proposed framework.

### 4.1. Output of One Layer

In the proposed framework, a group of member clusterers runs in each layer. Assume *n* is the data point’s number in a dataset. Each clusterer will generate a n×n matrix called the Partitioning Matrix (PM), where PM(i,j)=1 if the *i*-th and the *j*-th data points belong to the same cluster, or zero if otherwise. Assume *m* is the clusterer’s number. In each layer, we can obtain *m* instances of PM.

By extracting the value in position (i,j) from each PM, we can obtain a vector with the length *m* indicating the *m* clusterers’ judgments on whether the *i*-th and the *j*-th data points belong to the same cluster. We call this vector the Judgment Vector (JV).

Each pair of data points can generate one judgment vector, therefore, the outputs of one layer will be n(n−1)/2 instances of JV (the vectors generated for (i,j) and (j,i) are the same, and any data point must be in the same cluster with itself).

### 4.2. Identification of “Bad Data Points”

The “bad data points” refers to those data points that cannot be easily clustered correctly, e.g., the data points in the overlapping regions of two clusters. One possible pair of “bad data points” can be characterized by one JV that has elements with high discrepancy. It indicates that the clusterers have very different judgments on whether this pair of data points belongs to the same cluster, which means that these two data points might be hard to cluster correctly under the available conditions.

The discrepancy of the elements in one JV can be quantified by Shannon entropy as:

(9) $$H\left(JV\right)=-[\frac{m-\Delta }{m}\log_{2}\left(\frac{m-\Delta }{m}\right)+\frac{\Delta }{m}\log_{2}\left(\frac{\Delta }{m}\right)]$$

where Δ is the number of “1” elements in JV.

By calculating the entropy of each JV output from the previous layer, we can obtain an n×n matrix where the element in position (i,j) indicates the quantified discrepancy of the clusterers’ judgments on data points pair (i,j). We call this matrix the Entropy Matrix (EM). Assume *W* is a data point. In the dataset, there are *n* different pairs of data points that contains *W*. In these *n* pairs of data points, if the number of the pairs that have relatively high discrepancy is relatively large, then there will be a higher possibility that *W* is a “bad data point”.

Therefore, we define data point *W* to be a “bad data point” if:

(10) Card[high_En(W,α)]≥βn,α∈(0,1),β∈(0,1)

where Card[] returns the elements’ number in a set, β is a user-defined parameter and high_E(W,α) finds the elements in the *w*-th row (*w* is the order number of data point *W* in the *n* data points) of EM whose values are bigger than another user-defined parameter α.

In Equation (10), α and β are two parameters influencing the number of “bad data points” identified in a certain layer. The larger α and β are, the less “bad data points” will be found.

### 4.3. Output of the Clustering Ensemble Framework

After the “bad data points” are found in the previous layer, the clusterers in a new layer can generate new PMs based on different closeness measures. In this new layer, the clusterers will apply the CMNC metric when calculating the “distance” between the clusters’ centroids and the “bad data points”.

After obtaining the new PMs, we can further obtain the new JVs and the new EM. The “bad data points” identification algorithm will run again in this new layer and output the identified “bad data points” to the next new layer. This iteration stops when no more “bad data points” are found in a certain layer, or the user-given maximum iteration number *T* is met.

One additional instance of clustering methods will be executed using different closeness measures based on the finally found “bad data points” and normal data points to generate the final clustering results.

Following, we give an outline of the proposed clustering ensemble framework (See Algorithm 2).
**Algorithm 2**Input: *n* data points, number of clusterers *m*, max clusters number $k_{max}$, max iteration number *T*, parameters α, β.Output: data partitioningSteps:  S1. Initialize *m* member clusterers (*k*-medoids) with random *k* values (constrained to $k_{max}$) and random *k* clusters centroids.  S2. Calculate $PM_{1}$ to $PM_{m}$ with the *m* clusterers. If this is not the first time entering S2, then CMNC is applied to the “bad data points”. Otherwise, only Euclidean distance is used.  S3. Extract JV for every pair of data points from $PM_{1}$ to $PM_{m}$.  S4. Calculate information entropy on each JV, and generate EM.  S5. Identify “bad data points” based on EM.  S6. If no more “bad data points” are identified compared with the last iteration, or the iteration number reaches *T*, GO TO S7. Else, enter a new layer and GO TO S1.  S7. Generate the partitioning on the dataset. Return the clusters found.

### 4.4. Experiments

In this section, we will test the proposed clustering ensemble framework.

#### 4.4.1. Influence of Parameter Selection

In the proposed framework, parameters that need to be assigned by the user include the number of member clusterers *m*, the number of max clusters $k_{max}$, number of max iterations *T* and the parameters α, β.

The settings of *m*, $k_{max}$ and *T* do not significantly influence the clustering results, while α and β are two major parameters that influence the identification of “bad data points”. In the following, we fist examine how the two parameters can influence the “bad data points” detection. The test is run on the “double-moon” dataset, which contains two clusters and 400 data points.

**Figure 13:**
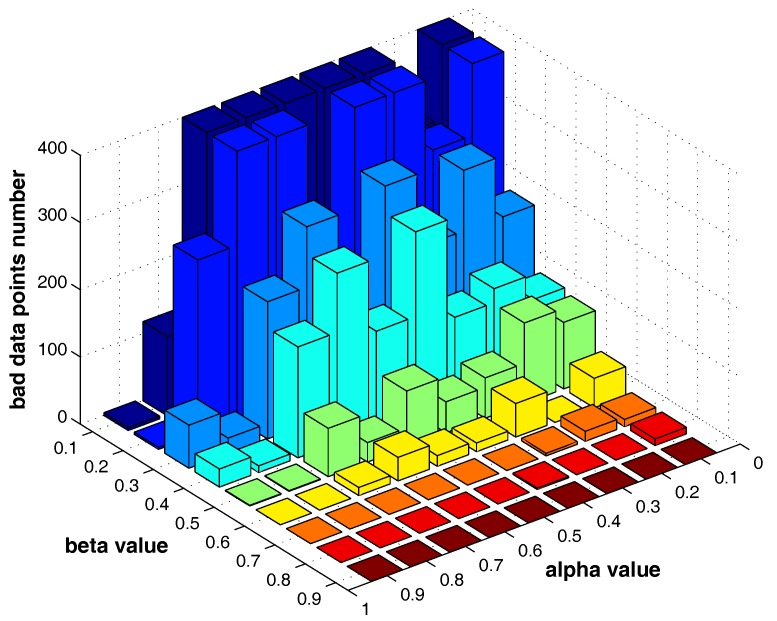
The influence of α and β on the “bad data points” identification, *m* = 5.

**Figure 14:**
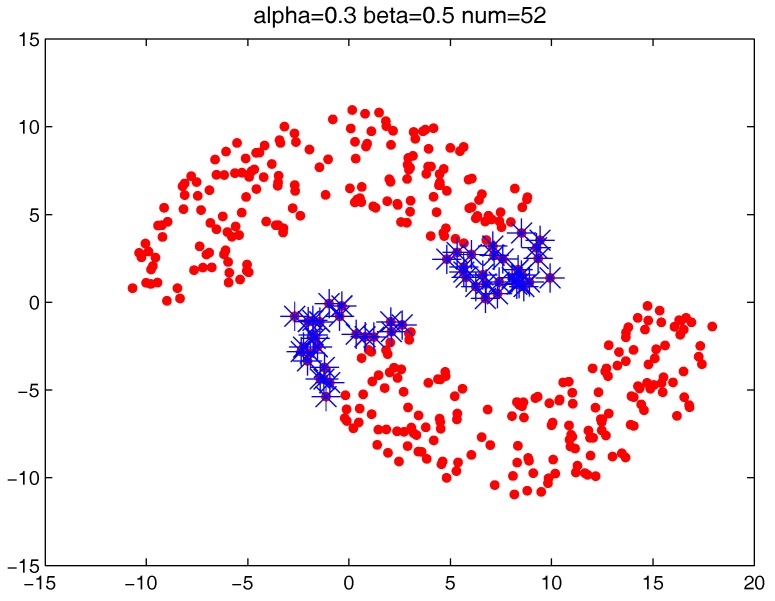
The “bad data points” found under α = 0.3, β = 0.5 and *m* = 5.

**Figure 15:**
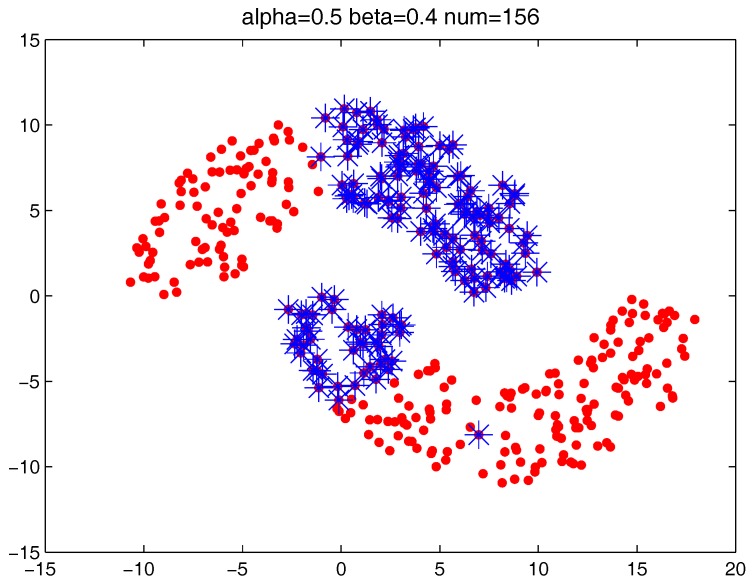
The “bad data points” found under α = 0.5, β = 0.4 and *m* = 5.

**Figure 16:**
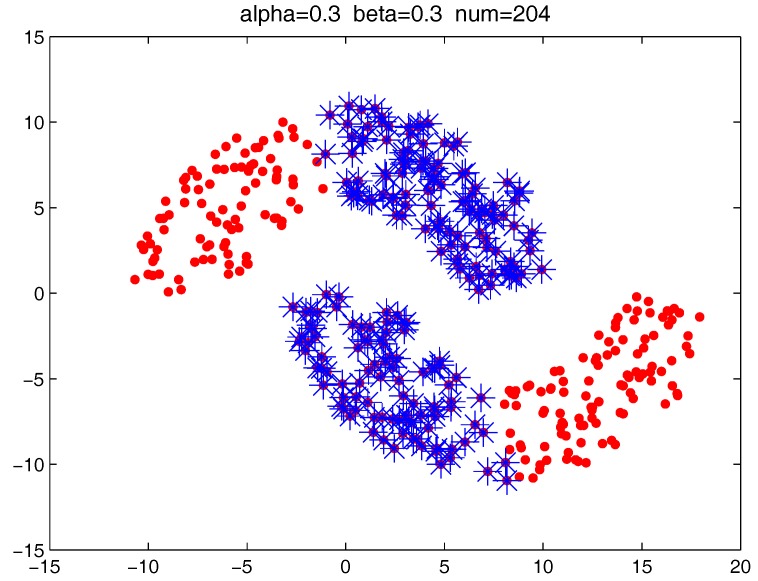
The “bad data points” found under α = 0.3, β = 0.3, and *m* = 5.

Figure 13 shows the number of the “bad data points” found by the identification algorithm under different α and β combinations. Figure 14, Figure 15 and Figure 16 locate the “bad data points” (the blue asterisks) found under three certain parameter combinations. The figures illustrate that along with the increase of the number of “bad data points” found, the remaining data points (the dots) become much more easier to cluster correctly with simple clustering algorithms.

**Figure 17:**
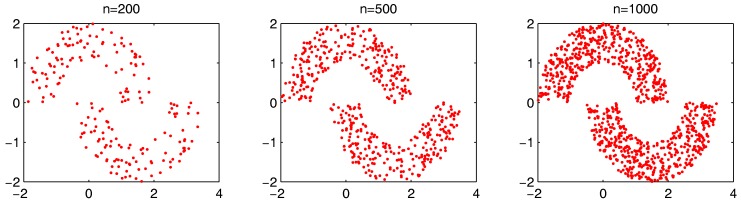
Synthetic dataset with different data numbers from 200 to 1000.

**Figure 18:**
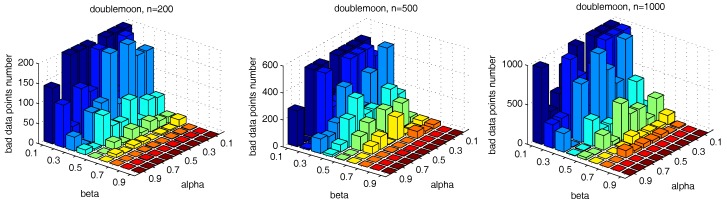
Number of “bad data points” found under different α and β combinations in datasets with different data numbers.

In order to more generally examine the effect of parameters α and β, we further test the parameter combinations on several more datasets. We first change the data point’s number in the “double-moon” dataset, varying from 200–1000, which is shown in Figure 17. The numbers of the “bad data points” found in these datasets under different α and β combinations are shown in Figure 18.

**Figure 19:**
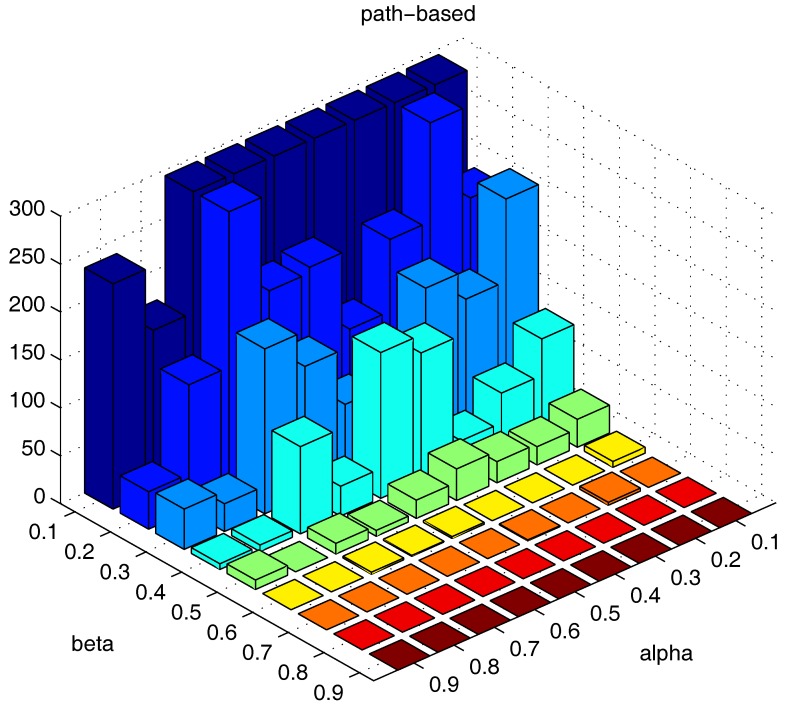
Number of “bad data points” found under different α and β combinations in the “path-based” dataset.

We also test the parameter combinations on another synthetic dataset “path-based” (the dataset is shown in Figure 11), and the numbers of the “bad data points” found under different parameter combinations are shown in Figure 19.

The results in Figure 18 and Figure 19 show that, in different datasets, parameters α and β influence the number of the “bad data points” in similar ways. Therefore, the changing tendency of the “bad data points” number presented under different parameter combinations illustrated in the figures can be seen as a general guide for the tested datasets when choosing the parameters in practice.

#### 4.4.2. Clustering Results of the Proposed Framework

In this section, we give the clustering results of the proposed clustering ensemble framework on some 2D datasets. The corresponding parameter settings are provided for each case.

**Figure 20:**
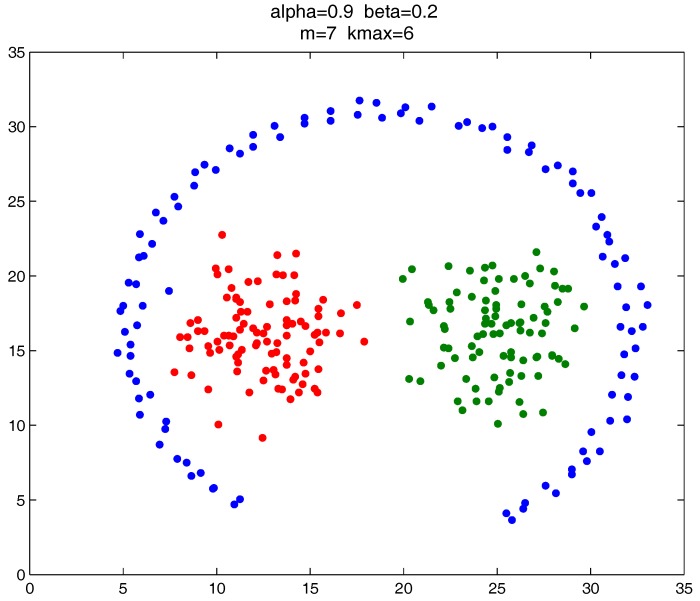
Clustering result on the “path-based” dataset.

**Figure 21:**
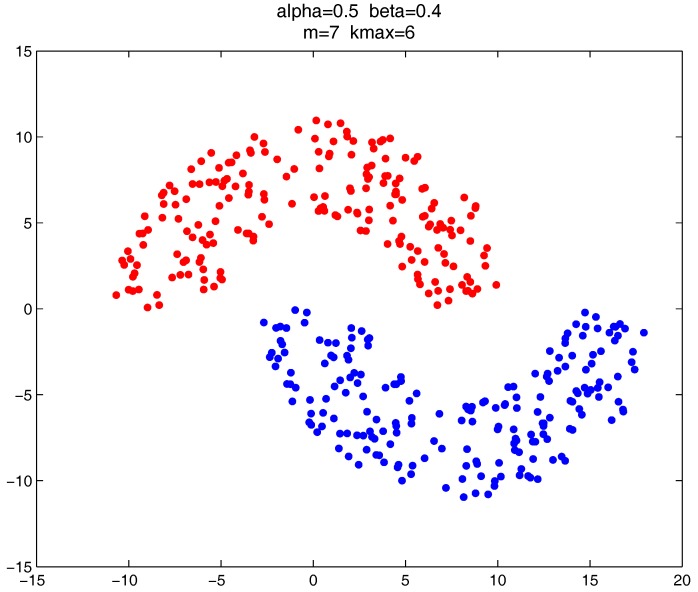
Clustering result on “double-moon” dataset.

**Figure 22:**
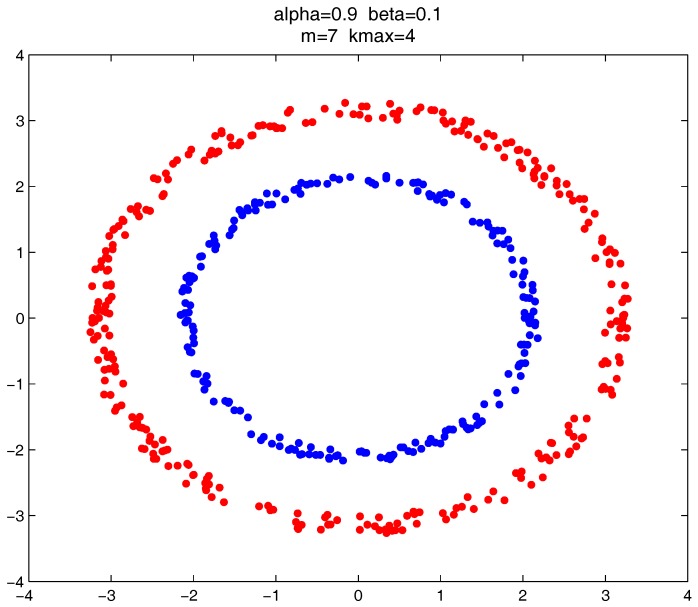
Clustering result on the “rings” dataset.

As shown in Figure 20, Figure 21 and Figure 22, all the tested datasets have non-spherically-shaped clusters. These datasets usually cannot be correctly clustered with the simple centroid-based clustering methods, or the clustering ensemble methods based on them. However, by applying the CMNC metric to part of the data points in the datasets, our proposed clustering ensemble framework can generate very good results on these datasets.

#### 4.4.3. Time Complexity of the Proposed Framework

Although using CMNC for the “bad data points” in our proposed framework will promote the accuracy of the clustering results, it is to some degree at the price of increasing the time complexity. Here, we will make a comparison of the execution time between the proposed framework combining different closeness measures and the traditional framework using only the Euclidean distance. The execution time of the method that directly uses CMNC to substitute the Euclidean distance in the traditional framework (i.e., the method without the “bad data points” identification) is also given for comparison. Meanwhile, we will also compare the execution time of the new framework under different parameter settings. All of the following tests are implemented in MATLAB R2009b, Microsoft Windows 10 operation system and based on Intel core i7 3.6-GHz CPU (quad core), 8 G DDR3 RAM.

**Figure 23:**
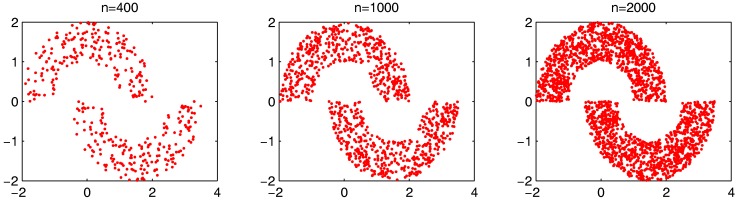
Synthetic dataset with different data numbers from 400–2000.

The factors that influence the computation time of the proposed framework include the total data points number *n*, the clusterers number *m* and the number of the found “bad data points” determined by α and β. First, we will examine the change of the computation time on the synthetic dataset “double-moon” when *n* increases. The dataset with different numbers of data points *n* is shown in Figure 23.

**Figure 24:**
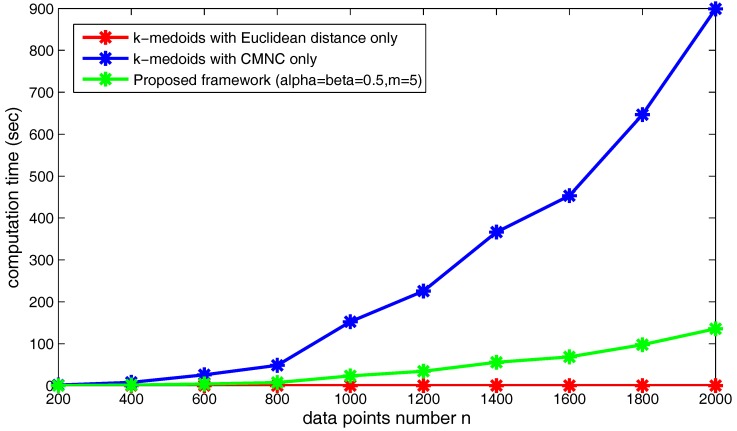
Increase of execution time when *n* increases.

Figure 24 shows the increasing of the execution time of the three kinds of methods when the data point’s number *n* increases. Obviously, under the shown parameter settings, the proposed framework saves much time compared to using CMNC only in the clustering method.

**Figure 25:**
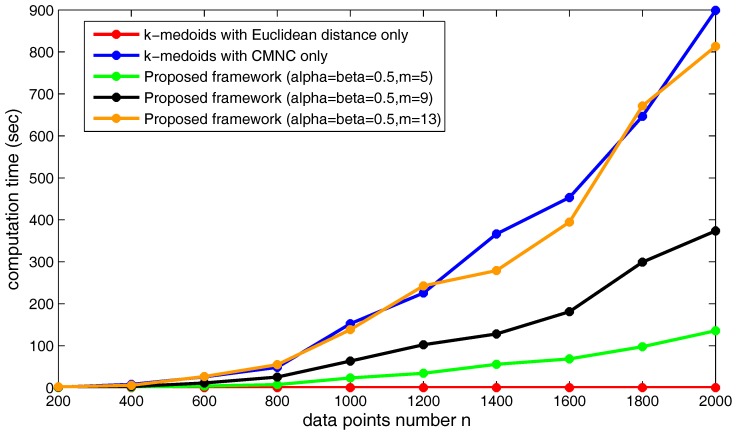
Increase of execution time when *m* increases.

Next, we will examine the change of the computation time when the clusterer’s number *m* changes. In Figure 25, the computation time of the proposed framework using different numbers of sub-clusterers (*m*) is illustrated. As shown in the figure, the computation time increases along with the increasing of *m*. Under the shown parameter settings, if *m* continues to grow, the computation time of the proposed framework will exceed the time cost by the clustering method using CMNC only. In practice, *m* could be assigned a relatively small value in order not to produce too much computational cost.

**Figure 26:**
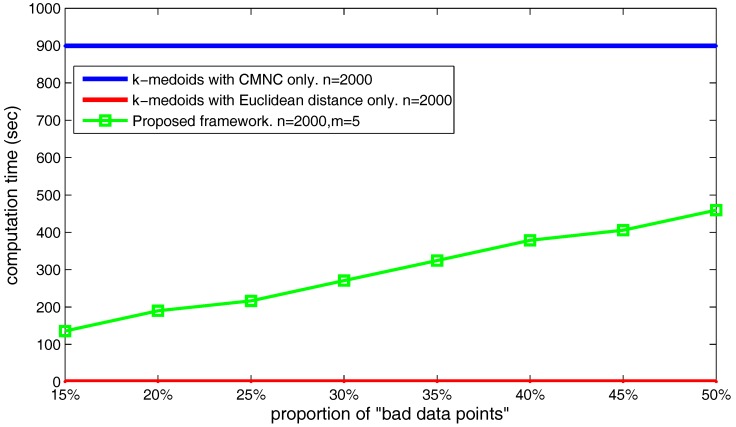
Increase of execution time when the number of the found “bad data points” increases.

The number of the found “bad data points” can also influence the computation time of the proposed framework. As shown in Figure 26, we set the data point’s number n=2000 and m=5, and choose different α, β values making the number of the found “bad data points” occupy from 20–50% of the total data point’s number. The figure shows that the time cost basically increases linearly with the increasing of the found “bad data points” number.

Generally speaking, the proposed framework can generate correct clustering results on the tasks with which the traditional ones cannot deal. By using the Euclidean distance and the CMNC measure together, the actual usage frequency of CMNC can be greatly lowered. Therefore, the framework is also much more time efficient than the method using CMNC only.

## 5. Conclusions

This paper proposes a novel closeness measure between data points based on the neighborhood chain called CMNC. Instead of using geometric distances alone, CMNC measures the closeness between data points by quantifying the difficulty to establish a neighborhood chain between the two points. As shown in the experimental results, by substituting the closeness measure in traditional clustering methods, the CMNC-based methods can achieve much better clustering results, especially in clustering tasks with arbitrary cluster shapes and different cluster scales.

Based on CMNC, we also propose a multi-layer clustering ensemble framework that combines two closeness measures: the Euclidean distance and the CMNC metric. A “bad data points” (data points that cannot be easily grouped into the correct cluster, e.g., the data points in the overlapping regions of the two non-spherically-shaped clusters) identification algorithm is proposed to find those data points whose closeness to the clusters’ centroids need to be computed with the CMNC metric. By incorporating the two closeness measures, the proposed framework can counter-act the drawbacks of the traditional clustering methods using the Euclidean distance alone. Meanwhile, it is more time efficient than the clustering method using the CMNC metric alone.

The major focus of our future work is to further reduce the computational complexity of the proposed framework. In our work, the execution time can be reduced by limiting the number of data points that use CMNC metric. However, we find that in some complicated cases, in order to ensure a tolerable clustering accuracy, a large proportion of data points will be identified to be the “bad data points”. This might lead to a significant decline in the time efficiency of the framework. To resolve the problem, more work should be done to optimize the nearest neighbors’ searching process (which produces the most computational cost in CMNC) and to further refine the “bad data points” identification algorithm. One possible improvement on which we will do further research is to find some key data points that can represent part or even the whole group of the “bad data points”. Applying CMNC on the representative data points might reduce the actual computation involving CMNC.

## Figures and Tables

**Table 1:** Clustering Error (CE) results of the tested methods.

	*k*-Medoids	CMNC-Based *k*-Medoids	Single-Link	CMNC-Based Single-Link	CURE	CMNC-Based CURE
iris	16.67%	14.67%	32.67%	32.00%	32.67%	30.67%
glass	43.27%	40.19%	62.62%	61.68%	59.81%	52.34%
wine	30.11%	28.65%	56.74%	41.57%	35.39%	31.46%
DataUser Modeling	47.09%	36.43%	63.57%	63.18%	64.73%	51.16%

**Table 2:** Normalized Mutual Information (NMI) results of the tested methods.

	*k*-Medoids	CMNC-Based *k*-Medoids	Single-Link	CMNC-Based Single-Link	CURE	CMNC-Based CURE
iris	0.6957	0.7151	0.7355	0.7452	0.7229	0.7452
glass	0.3148	0.3668	0.1196	0.1415	0.1751	0.3523
wine	0.4199	0.4326	0.0914	0.3041	0.3982	0.4392
DataUser Modeling	0.2316	0.3173	0.0774	0.0822	0.0490	0.1098

**Table 3:** Significance test of CE results: CMNC-based *k*-medoids vs. *k*-medoids.

	*t*-Statistic Value	Rejection Region ( α=0.1)	$H_{0}$: $\mathrm{mean}\left(CE_{\mathrm{CMNC}-\mathrm{based}-k-\mathrm{medoids}}\right)=\mathrm{mean}\left(CE_{k-\mathrm{medoids}}\right)$
iris	−2.5131	$\left|t\right|>1.6860$	rejected
glass	−3.0482	$\left|t\right|>1.6860$	rejected
wine	−1.8074	$\left|t\right|>1.6860$	rejected
DataUser Modeling	−2.9970	$\left|t\right|>1.6860$	rejected

**Table 4:** Significance test of NMI results: CMNC-based *k*-medoids vs. *k*-medoids.

	*t*-Statistic Value	Rejection Region (α=0.1)	$H_{0}$: $\mathrm{mean}\left(\mathrm{NM}I_{\mathrm{CMNC}-\mathrm{based}-k-\mathrm{medoids}}\right)=\mathrm{mean}\left(\mathrm{NM}I_{k-\mathrm{medoids}}\right)$
iris	2.8505	$\left|t\right|>1.6860$	rejected
glass	7.5803	$\left|t\right|>1.6860$	rejected
wine	3.7374	$\left|t\right|>1.6860$	rejected
DataUser Modeling	3.6979	$\left|t\right|>1.6860$	rejected

## References

[1] Bhatti D.M.S., Saeed N., Nam H. (2016). Fuzzy C-Means Clustering and Energy Efficient Cluster Head Selection for Cooperative Sensor Network. Sensors.

[2] Tang C., Shokla S.K., Modhawar G., Wang Q. (2016). An Effective Collaborative Mobile Weighted Clustering Schemes for Energy Balancing in Wireless Sensor Networks. Sensors.

[3] Mammu A.S.K., Hernandez-Jayo U., Sainz N., Iglesia I.D.L. (2015). Cross-Layer Cluster-Based Energy-Efficient Protocol for Wireless Sensor Networks. Sensors.

[4] Jiang P., Liu J., Wu F. (2015). Node Non-Uniform Deployment Based on Clustering Algorithm for Underwater Sensor Networks. Sensors.

[5] Liu G.Y., Zhang Y., Wang A. (2015). Incorporating Adaptive Local Information Into Fuzzy Clustering for Image Segmentation. IEEE Trans. Image Proc..

[6] Wang L.F., Pan C.H. (2014). Robust level set image segmentation via a local correntropy-based K-means clustering. Pattern Recognit..

[7] Ji Z.X., Liu J.Y., Cao G., Sun Q.S., Chen Q. (2014). Robust spatially constrained fuzzy c-means algorithm for brain MR image segmentation. Pattern Recognit..

[8] Yang C.C., Ng T.D. (2011). Analyzing and Visualizing Web Opinion Development and Social Interactions With Density-Based Clustering. IEEE Trans. Syst. Man Cybern. Part A.

[9] Huang Q.H., Wang T., Tao D.C. (2015). Biclustering Learning of Trading Rules. IEEE Trans. Cybern..

[10] Mahmood A., Small M. (2016). Subspace Based Network Community Detection Using Sparse Linear Coding. IEEE Trans. Knowl. Data Eng..

[11] Cai X.Y., Li W.J. (2013). Ranking Through Clustering: An Integrated Approach to Multi-Document Summarization. IEEE Trans. Audio Speech Language Proc..

[12] Lin Y.S., Jiang J.Y., Lee S.J. (2014). A Similarity Measure for Text Classification and Clustering. IEEE Trans. Knowl. and Data Eng..

[13] MacQueen J.B. (1967). Some methods for classification and analysis of multivariate observations. Proc. 5th Berkeley Symp. Math. Statist. Probabil..

[14] Ester M., Kriegel H.P., Xu X. A density-based algorithm for discovering clusters in large spatial databases with noise. Proceedings of the Second International Conference on Knowledge Discovery and Data Mining.

[15] Guha S., Rastogi R., Shim K. (2001). Cure: An Efficient Clustering Algorithm for Large Databases. Inf. Syst..

[16] Tzortzis G., Likas A. (2014). The MinMax k-Means clusteringalgorithm. Pattern Recognit..

[17] Malinen M.I., Istodor R.M., Fränti P. (2014). K-means*: Clustering by gradual data transformation. Pattern Recognit..

[18] Kumar K.M., Reddy A.R.M. (2016). A fast DBSCAN clustering algorithm by accelerating neighbor searching using Groups method. Pattern Recognit..

[19] Morsier F.D., Tuia D., Borgeaud M., Gass V., Thiran J.P. (2015). Cluster validity measure and merging system for hierarchical clustering considering outliers. Pattern Recognit..

[20] Olszewski D., Ster B. (2014). Asymmetric clustering using the alpha-beta divergence. Pattern Recognit..

[21] Tabor J., Spurek P. (2014). Cross-entropy clustering. Pattern Recognit..

[22] Lu Y.G., Wan Y. (2013). PHA: A fast potential-based hierarchical agglomerative clustering method. Pattern Recognit..

[23] Chen L.F., Wang S.R., Wang K.J., Zhu J.P. (2016). Soft subspace clustering of categorical data with probabilistic distance. Pattern Recognit..

[24] Karypis G., Han E.H., Kumar V. (1999). Chameleon: A Hierarchical Clustering Using Dynamic Modeling. Computer.

[25] Jiang B., Pei J., Tao Y.F., Lin X.M. (2013). Clustering Uncertain Data Based on Probability Distribution Similarity. IEEE Trans. Knowl. Data Eng..

[26] Jain A.K., Dubes R.C. (1988). Algorithms for Clustering Data.

[27] Omran M.G., Engelbrecht A.P., Salman A. (2007). An overview of clustering methods. Intell. Data Anal..

[28] Lin C.R., Chen M.S. (2005). Combining Partitional and Hierarchical Algorithms for Robust and Efficient Data Clustering with Cohesion Self-Merging. IEEE Trans. Knowl. Data Eng..

[29] Dhillon I.S., Maella S., Kumar R. Enhanced word clustering for hierarchical text classification. Proceedings of the ACM SIGKDD International Conference on Knowledge Discovery and Data Mining.

[30] Kullback S., Leibler R.A. (1951). On Information and Sufficiency. Ann. Math. Stat..

[31] Dhillon I.S., Maella S., Kumar R. (2003). A divisive information-theoretic feature clustering algorithm for text classification. J. Mach. Learn. Res..

[32] Dhillon I.S., Maella S., Kumar R. Information-theoretic co-clustering. Proceedings of the ACM SIGKDD International Conference on Knowledge Discovery and Data Mining.

[33] Heller K.A., Ghahramani Z. Bayesian hierarchical clustering. Proceedings of the 22nd International Conference on Machine Learning.

[34] Teh Y.W., Lii H.D., Roy D. Bayesian Agglomerative Clustering with Coalescents. Proceedings of the International Conference on Neural Information Processing Systems.

[35] Grady L., Schwartz E. (2006). Isoperimetric graph partitioning for image segmentation. IEEE Trans. Pattern Anal. Mach. Intell..

[36] Pavan M., Pelillo M. (2007). Dominant sets and pairwise clustering. IEEE Trans. Pattern Anal. Mach. Intell..

[37] Zhang W., Zhao D.L., Wang X.G. (2013). Agglomerative clustering via maximum incremental path integral. Pattern Recognit..

[38] Liu Y.H., Ma Z.M., Yu F. (2017). Adaptive density peak clustering based on K-nearest neighbors with aggregating strategy. Knowl.-Based Syst..

[39] Rodriguez A., Laio A. (2014). Clustering by fast search and find of density peaks. Science.

[40] Sur A., Chowdhury A., Chowdhury J.G., Das S. Automatic Clustering Based on Cluster Nearest Neighbor Distance (CNND) Algorithm. Proceedings of the International Conference on Frontiers of Intelligent Computing: Theory and Applications (FICTA).

[41] Qiu T., Yang K., Li C., Li Y. A Physically Inspired Clustering Algorithm: To Evolve Like Particles. https://arxiv.org/abs/1412.5902.

[42] Qiu T., Li Y. Clustering by Descending to the Nearest Neighbor in the Delaunay Graph Space. https://arxiv.org/abs/1502.04502.

[43] Qiu T., Li Y. IT-Dendrogram: A New Member of the In-Tree (IT) Clustering Family. https://arxiv.org/abs/1507.08155.

[44] Qiu T., Li Y. Clustering by Deep Nearest Neighbor Descent (D-NND): A Density-based Parameter-Insensitive Clustering Method. https://arxiv.org/abs/1512.02097.

[45] Luo C., Li Y., Chung S.M. (2009). Text document clustering based on neighbors. Data Knowl. Eng..

[46] Nie F., Wang X., Huang H. Clustering and projected clustering with adaptive neighbors. Proceedings of the 20th ACM SIGKDD international conference on Knowledge discovery and data mining.

[47] Liang S.Y., Han D.Q., Zhang L., Peng Q.K. A Novel Clustering Oriented Closeness Measure Based on Neighborhood Chain. Proceedings of the 2017 International Joint Conference on Neural Networks.

[48] Kaufmann L., Rousseeuw P.J. (1987). Clustering by Means of Medoids. Statistical Data Analysis Based on the L1-norm & Related Methods.

[49] Sneath P., Sokal R. (1973). Numerical Taxonomy.

[50] Ankerst M., Breunig M.M., Kriegel H.P., Sander J. (1999). OPTICS: Ordering Points To Identify the Clustering Structure. ACM Sigmod Rec..

[51] Jain A., Law M. (2005). Data clustering: A user’s dilemma. Comput. Sci..

[52] Zahn C.T. (1971). Graph-theoretical methods for detecting and describing gestalt clusters. IEEE Trans. Comput..

[53] Chang H., Yeung D.Y. (2008). Robust path-based spectral clustering. Pattern Recognit..

[54] Blake C., Merz C.J. (1998). UCI Repository of Machine Learning Databases. http://www/ics.uci.edu/mlearn/MLRepository.html.

[55] Strehl A., Ghosh J. (2002). Cluster Ensembles—A Knowledge Reuse Framework for Combining Multiple Partitions. J. Mach. Learn. Res..

[56] Wu M.R., Scholköpf B. A Local Learning Approach for Clustering. Proceedings of the 20th Annual Conference on Neural Information Processing Systems.

[57] Gosset W.S. (1908). The Probable Error of a Mean. Biometrika.

